# Progress in Child and Adolescent Psychiatry and Mental Health

**DOI:** 10.3390/children12030274

**Published:** 2025-02-24

**Authors:** Florina Rad

**Affiliations:** 1 Child and Adolescent Psychiatry Department, “Carol Davila” University of Medicine and Pharmacy, 020021 Bucharest, Romania; florina.rad@umfcd.ro; 2 Child and Adolescent Psychiatry Department, “Prof. Dr. Al. Obregia” Clinical Hospital of Psychiatry, 041914 Bucharest, Romania

## 1. Introduction

Most mental health conditions emerge in early childhood and their prevalence varies with age. At a global scale, mental illnesses are thought to be the main contributors to the burden of disease in children and adolescents. Based on the results of a recent study, one in seven patients between the ages of 3 and 17 (13%) had a mental or behavioral health issue that had been formally diagnosed [1]. The fact that some of these mental health disorders frequently co-occur presents an even more worrisome picture. More than one in three (37%) children with a mental health condition in 2018–2019 had two or more comorbid psychiatric diagnoses [1]. It is crucial to remember that a child’s mental health status cannot be fully explained by a diagnosis, the reason being that children may exhibit certain symptoms without fulfilling all of the required diagnosis criteria, or they may meet the requirements for a diagnosis but remain undiagnosed or untreated by mental health specialists.

A significant number of depressive episodes emerge in childhood and their frequency increases during adolescence [2]. Research shows that 25% of teenagers have had a depressive episode by the time they reach 19 years of age [3]. The onset of depression in adolescence is frequently associated with low quality of life, deficits in interpersonal relationships, academic and professional setbacks, increased rates of physical and mental illness, relapses, and an increased risk of suicide and self-harm injuries.

Over the past ten years, there have been significant advancements in the diagnosis of neurodevelopmental disorders, particularly attention-deficit/hyperactivity disorder (ADHD) and autism spectrum disorder (ASD). The number of children with ASD has increased globally in recent decades. This increase is thought to be caused by a combination of actual increases in ASD cases and a better understanding of the disorder and diagnostic techniques.

The following Special Issue includes 12 articles presenting a comprehensive exploration of current research and clinical advancements in neurodevelopmental and emotional disorders affecting children and adolescents. The articles cover key topics such as autism spectrum disorder (ASD) and attention-deficit/hyperactivity disorder (ADHD), highlighting new insights into diagnosis, intervention strategies, and neurobiological aspects. Additionally, this Special Issue addresses pediatric depression, examining risk factors, treatment approaches, and emerging biomarkers that may help in the early identification of internalization disorders. The impact of the COVID-19 pandemic is another focal point, with studies investigating post-pandemic family functionality, shifts in adolescent emotional regulation, and broader psychosocial effects on youth mental health.

By releasing the Special Issue “Child and Adolescent Psychiatry and Mental Health Progress”, we intend to disseminate new scientific discoveries regarding the continuous challenges specialists face and enhance mental health. The contributions included in this Special Issue provide crucial information for advanced research and address the unique mental health challenges faced by younger populations. Children and adolescents experience mental health difficulties that can differ significantly from those of adults; early intervention in this regard can profoundly impact long-term outcomes. This dedicated Special Issue facilitates an in-depth exploration of emerging trends, innovative treatments, and effective measures in this field. The editorial team hopes that the journal *Children* succeeds in providing a platform for sharing diverse perspectives and findings that can inform both clinical practice and research specialists. By highlighting the importance of mental health in children and adolescents, published scientific contributions can raise awareness, facilitate collaboration, and promote the development of evidence-based approaches that improve the overall well-being of children and adolescents globally.

## 2. Overview of the Published Articles

The data and findings presented in the published papers are highlighted as follows:

- The findings of Contribution 1 indicate that teenagers with ADHD symptoms may still face notable difficulties in connection with these symptoms even if they do not have a formal diagnosis. Relationships between ADHD symptoms and mental health were found to be significantly mediated by three peer relationship variables: perceived isolation, friendships, and sense of belonging.
- The authors of Contribution 2 examine family dynamics and their contribution to adolescent depression diagnosis. The results of this study highlight a comprehensive approach to addressing teenage depression, which includes cognitive flexibility, family-based assessment, and screening for maternal depressive symptoms.
- The authors of Contribution 3 describe the cognitive–emotional regulation strategies used by adolescents with major depressive disorder, with the results demonstrating that using maladaptive coping mechanisms (such as catastrophizing and rumination) and adaptive coping mechanisms (such as positive reappraisal) are adversely correlated with depression.
- The authors of Contribution 4 assess the challenges faced by adolescents after the COVID-19 pandemic in terms of loneliness, social withdrawal, and personality functioning. The results demonstrate the direct effect of these key factors on mental health issues in adolescents and the need for admittance to an acute psychiatric unit.
- The authors of Contribution 5 describe the effects of COVID-19 pandemic restrictions on family communication, functioning, and resilience in Algeria and Iraq, with the results showing that family communication mediates the relationship between family resilience and family functioning.
- An evaluation of the trajectory of internalizing/externalizing symptoms or the association between these factors and the family environment in children with Williams syndrome is presented in Contribution 6. Based on CBCL profiles, the prevalence of internalizing and externalizing issues continued to be persistently high over time in this category of patients. The family environment exhibited no significant correlation with the evolution of internalizing/externalizing symptoms.
- Children and adolescents with self-regulation difficulties (internalization and externalization problems) and executive dysfunctions present impaired functionality in daily activities and lower quality of life. These aspects are investigated in Contribution 7.
- Contribution 8 is a retrospective review and evaluates the characteristics of comorbidities among children diagnosed with ASD, with the results indicating high rates of comorbidities in the ASD group, with the most frequently encountered being neurological associations.
- The authors of Contribution 9 aimed to assess the social determinants of resilience in the mental health of Chilean adolescents, with the results pointing to an increased prevalence of depression and anxiety symptoms in the female teenage study group.
- The authors of Contribution 10 highlight potential biological markers linked to physiological responses in adolescents diagnosed with major depressive disorder during parental conflict. Elevated heart rate or lower respiratory sinus arrhythmia could represent physiological factors used in order to assess the emotional impacts of parental conflict on a child diagnosed with depression.
- The authors of Contribution 11 examine the efficiency of psychosocial intervention techniques in the hyperactive/impulsive form of ADHD in children. Psychoeducation and training for parents, school-based interventions, reinforcement strategies, and neurofeedback consistently showed small-to-moderate effect sizes in reducing hyperactivity/impulsivity in children.
- The authors of Contribution 12 present the case of a 10-year-old girl with anorexia nervosa comorbid with ADHD. The novelty and uniqueness of the reported case consisted of the hypothesis that the induced weight loss might suppress ADHD symptoms.

## 3. Acknowledgments and Conclusions

We extend our deepest gratitude to the authors whose valuable contributions have enriched this Special Issue. Their dedication to scientific inquiry and commitment to advancing knowledge have played a pivotal role in shaping the discourse presented herein. We also wish to express our sincere appreciation to our editorial team and peer reviewers. Their collective expertise has ensured the integrity and quality of the included publications.

We hope that the perspectives presented in the published articles stimulate new directions for future research.
